# Role of active and environmental tobacco smoke on susceptibility to osteoporosis in women undergoing dual-X-ray absorptiometry

**DOI:** 10.1007/s40618-023-02211-3

**Published:** 2023-10-11

**Authors:** A. Vergatti, V. Abate, A. Giaquinto, N. Altavilla, L. D’Elia, M. Evangelista, G. De Filippo, G. Piccinocchi, L. Gennari, D. Merlotti, F. Galletti, P. Strazzullo, D. Rendina

**Affiliations:** 1 Department of Clinical Medicine and Surgery, Federico II University, Naples, Italy; 2 Assistance Publique—Hôpitaux de Paris, Hôpital Robert-Debré, Service d’Endocrinologie et Diabétologie, Paris, France; 3 “COMEGEN” Medical Cooperative, Naples, Italy; 4 Department of Medicine, Surgery and Neurosciences, University of Siena, Siena, Italy; 5 Tobacco Treatment Center, Department of Clinical Medicine and Surgery, Federico II University, Naples, Italy

**Keywords:** Smoking habits, Environmental tobacco smoke, Bone mineral density, Osteoporosis, Cross-sectional study, Administrative data

## Abstract

**Purpose:**

Current smoking is a risk factor for osteoporosis (Op), but few data are available regarding the passive smoke impact on Op susceptibility. This cross-sectional study aimed to evaluate the association between the smoking habits and Op in community-dwelling women undergoing dual-energy X-ray absorptiometry (DXA).

**Methods:**

On 01/06/2018, general practitioners from “COMEGEN” Medical Cooperative, Naples, Italy, selected the medical records from the last 10 years of women who had a measurement of bone mineral density performed and simultaneously completed a questionnaire about their smoking behaviour and their cohabiters’. The binary logistic regression analysis was used to estimate the role of passive smoke on the risk of Op, adjusting for age and body mass index (BMI).

**Results:**

Among 10,616 subjects, 3942 were currently smokers [CS; mean age 69.4 ± 10.4 years; BMI 27.0 ± 4.9 kg/m^2^], 873 were passive smokers (PS; mean age 67.8 ± 11.6 years; BMI 27.0 ± 4.9 kg/m^2^) and 5781 were never smokers (NS; mean age 67.8 ± 11.6 years; body mass index (BMI) 27.0 ± 4.9 kg/m^2^). Of all, 8562 women (mean age 70.3 ± 10.2 yrs; BMI 27.0 ± 4.9 kg/m^2^) received the Op diagnosis. PS showed an increased Op risk compared to NS [odds ratio (OR) 1.38 (1.14–1.67)] and comparable to CS [OR 1.02 (0.84–1.24)].

**Conclusion:**

The study results demonstrate an association between passive smoke and Op in community-dwelling women already presenting with susceptibility to Op according to Italian essential assistance levels, suggesting that passive and active smoke are equivalent Op risk factors in women.

## Introduction

Smoke has a detrimental impact on human health, either as active or as passive smoking. It poses heavy health- and economic-related consequences worldwide [1–3]. Environmental tobacco smoke (ETS), also known as passive smoke, is defined by the World Health Organization (WHO) includes materials in the air originating from burned or heated tobacco. Exposure continues after smoking stops and includes second- and third-hand smoke. Second-hand smoke includes smoke released by actively burning tobacco products, while third-hand smoke refers to particles re-suspended in air. Sources include furniture, clothing, and smokers’ breath [4, 5].All forms of ETS are detrimental to health. Children are at particular risk, not being able to control their environments and re-volatizing third-hand smoke particles through active play and exploration [4, 5]. Smoke is a well-known risk factor for complex and multifactorial disorders, including lung, breast, and cervical cancers, cardiovascular diseases, and dementia [6–9]. It is currently responsible for over 600,000 deaths every year and the large majority of passive smoking deaths occur prematurely [5]. Based on these dramatic data, the WHO took a close focus on the smoking prevention [10] as well as the Italian National Institute of Health (in Italian, *Istituto Superiore di Sanità*, ISS) that, considering the WHO directives, published the guidelines for the prevention of health damage related to second-hand smoke [11].

Osteoporosis (Op) is the most common chronic metabolic bone disease worldwide and it is clinically defined by a reduced bone mineral density (BMD), estimated by dual-energy X-ray absorptiometry (DXA), resulting in increased risk of fragility fractures [12]. In 2010, Op affected 22 million European women and 5.5 million European men, and the economic burden of incidental and prior fragility fractures was estimated at € 37 billion [12, 13]. Considering the significant human and economic load of Op, the Italian Ministry of Health established a plan to prevent fragility fractures via the essential assistance levels, by determining the BMD by DXA in community-dwelling individuals, already presenting with an increased risk of Op, as: i) men and women of any age in the presence of a major risk factor (previous fragility fractures; radiological finding of osteoporosis; chronic therapies rising the risk of osteoporosis; diagnosed diseases, with higher risk of osteoporosis [14]), ii) post-menopausal women in the presence of a major risk factor (family history of bone fracture at age lower than 75 years; body mass index < 19 kg/m^2^; menopause at age lower than 45 years [14]), iii) postmenopausal women and men over the age of 60 years in the presence of at least three or more minor risk factors for Op (age > 65 years, family history of severe osteoporosis; amenorrhoea > 6 months in premenopausal age; inadequate calcium intake; vitamin D deficiency; smoking more than 20 cigarettes/day; alcohol abuse [> 60 g/day][14]).

Currently, cigarette smoking is already considered a significant Op risk factor [14], since it inversely correlates with BMD in both elderly men and women, in perimenopausal women, and even in young men [15]. However, few data are available regarding the association between ETS and Op, preventing national health-care systems from considering ETS as a real risk factor for Op. The aim of this cross-sectional study was to evaluate the Op prevalence in community-dwelling women current smoker (CS), passive smokers (PS) and never smokers (NS) of European ancestry, subjected to a DXA evaluation of BMD, according to Italian EALs. NS women were dichotomized into those exposed to PS and those not exposed to ETS according to smoking habits of their cohabitants. All enrolled patients were resident in the Naples 1 district of the National Health System (in Italian, *Azienda Sanitaria Locale, ASL, Naples 1*) in the Campania region (Italy), a geographic area with high environmental air pollution [16].

## Methods

This cross-sectional retrospective study was based on the clinical records of all patients followed by the general practitioners (GPs) referring to the “COMEGEN” *(COoperativa di MEdicina GENerale* in Italian) Medical Cooperative operating within the urban area of ASL Naples 1 (40°50′N 14°15′E). As of June 1, 2018, the participating GPs selected, among their patients, women who had simultaneously undergone an evaluation of BMD by DXA [International Classification of Diseases—9th revision (ICD9) code 8898] because of clinically suspected of Op according to the Italian Ministry of Health prevention plan and completed a questionnaire that included queries on their smoking behaviour and their cohabiter’s between June 1, 2008 and May 31, 2018. In case of more than one contextual assessment of BMD by DXA and of smoking habits questionnaire in the lapse of time considered, only the data referring to the first contextual assessment were collected. From all the medical records, other than DXA results and answers to smoking questionnaire, data regarding weight, height, waist circumference, body mass index (BMI), age, sex, estimated glomerular filtration rate (eGFR, estimated by a standard formula [17]), and any pharmacological treatments were collected as part of clinical practice. Since that, data regarding the number of daily cigarettes smoked were not available for this study. Informed consent was obtained from all individual participants included in the study, also as part of clinical practice. All data were collected in an electronic file. This study is part of the SIMON (metabolic syndrome, osteoporosis, and nephrolithiasis; *SIndrome Metabolica, Osteoporosi e Nefrolitiasi* in Italian) protocol [18–20], which was approved by the ASL Naples 1 Ethical Committee, protocol number 0018508/2018.

### Op diagnosis

The Op diagnosis was based on the following criteria: (1) T-score value measured by DXA ≤ − 2.5 in the lumbar spine, total hip or femoral neck, according to the WHO diagnostic criteria [21]; (2) fragility fractures, only diagnosed by radiograph images (ICD9 codes 73,310 to 73,319); (3) personal history of anti-osteoporotic treatment according to Italian Medicine Agency prescriptive criteria (in Italian, *Agenzia Italiana del FArmaco—*AIFA) [22]. Subjects with a lumbar, total hip or femoral T-score > − 2.5 and a personal history negative for clinical fragility fractures and anti-osteoporotic treatment were considered as not osteoporotic women.

### Smoking questionnaire and smoking habits classification

The smoking questionnaires included the following questions: (a) Have you ever smoked? Eventually, when did you initiate? (b) Have you ever smoked? Eventually, when did you stop? (c) Do your cohabitants or co-workers smoke in your presence? The smoking habits of the population were defined according to the smoking questionnaire replies. Firstly, the population was asked about current and lifetime smoking habits, smoking initiation, and eventual age at smoking cessation (question a and/or b). In case the reply to question b was positive and the subjects reported to have stopped smoking habits from more than 12 months, they were excluded from the study. Then, subjects that replied positively to question a were then classified as current smokers (CS), because of a regular consumption of cigarettes reported (duration > 6 months) at the time of examination; if not, as non-smokers. In case the subject was defined as CS, the last query was not administered. Instead, non-smokers were further asked if there were smokers living in the participant's family currently or during childhood or at workplace (question c). If the responses were affirmative to any of the previous questions, non-smokers were classified as PS. Otherwise, if subjects were non-smokers and they never had a smoker as relative or as a co-worker, the responses to all the questions were negative (a, b and c) and they were classified as never smokers (NS).

### Exclusion criteria

Pre-established exclusion criteria were all the known causes of secondary Op, apart from cigarette smoking, such as: age lower than 40 years, ever diagnosis of malabsorption syndromes (ICD9 codes 5793 to 5799), rheumatoid arthritis (ICD9 code 7140), long-term immobilization, moderate to severe chronic kidney disease [estimated glomerular filtration rate < 60 ml/min/1.73 m^2^ [17] (ICD9 codes 5853–5859, 586 and 6393)], hyperthyroidism (ICD9 codes 24,200–24291), primary hyperparathyroidism (ICD9 codes 25,200–25208), hypoparathyroidism (ICD9 code 2521), Cushing’s syndrome (ICD9 code 2550), chronic liver disease (ICD9 codes 5710–5719), pituitary tumours (ICD9 codes 1943, 2273, 2370), surgical history of terminal ileal resection (ICD9 code 4562), gastrectomy or small bowel bypass (ICD9 codes 430–4499), bilateral ovariosalpingectomy (ICD9 code 6551, 6553, 6561, 6563), eating disorders (ICD9 codes 3071 and 30,750–30759), alcoholism (ICD9 codes 30,390–30393), regular use of gonadotropin-releasing hormone agonist, glucocorticoids, anticonvulsants, heparin, vitamin A and cytotoxic agents for a current or past diagnosis of cancer. Subjects with history of Op treatment not compliant to AIFA prescriptive criteria were also excluded [22]. Past smokers were also excluded. This latter was defined as individuals who had smoked during their lifetime but not in the last 12 months, prior to the time of the baseline examination [23].

### Statistical analysis

Statistical analysis was performed using IBM SPSS (Statistical Package for Social Science), version 25 (IBM, Armonk, NY, USA). In univariate analyses, statistical comparisons were based on the Student’s *t* test for continuous variables and on the Chi-squared test for dichotomous variables. The binary logistic regression analysis was used to estimate the role of SHS on the risk of Op, adjusting for age and BMI. The collinearity among variables included in the models was assessed. The analysis did not detect any collinearity among variables (tolerance: 0.94, variance inflation factor 1.0). All statistical tests were two-tailed. The results are reported as mean (standard deviation – SD), or absolute, or percentages or as odds ratio (OR) and 95% confidence interval (95% CI). A *p* value < 0.05 was considered significant.

## Results

**Fig. 1:**
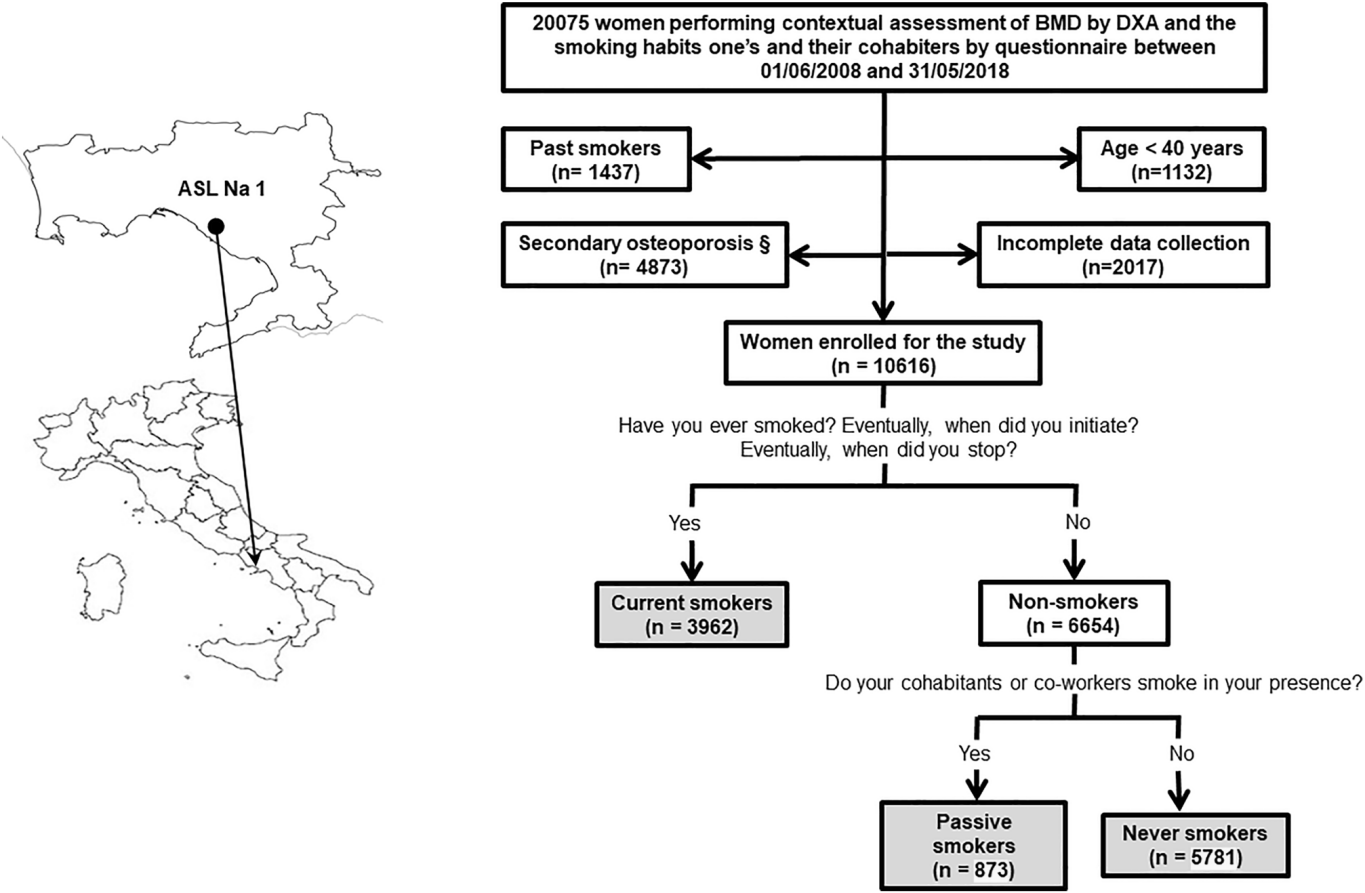
Study flowchart. The study population is highlighted in grey. ASL Na 1: Local Health Unit (in Italian: *Azienda Sanitaria Locale*) Naples 1. BMD: bone mineral density assessed by dual-energy X-ray absorptiometry. DXA: dual-energy X-ray absorptiometry. *§*: subjects with malabsorption syndromes [International Classification of Diseases-9th revision (ICD9) codes 5793 to 5799], rheumatoid arthritis (ICD9 code 7140), long-term immobilization, moderate to severe chronic kidney disease [estimated glomerular filtration rate < 60 ml/min/1.73 m2 [17] (ICD9 codes 5853–5859, 586 and 6393], hyperthyroidism (ICD9 codes 24,200–24291), primary hyperparathyroidism (ICD9 codes 25,200–25208), hypoparathyroidism (ICD9 code 2521), Cushing’s syndrome (ICD9 code 2550), chronic liver disease (ICD9 codes 5710–5719), bilateral ovariosalpingectomy (ICD9 code 6551, 6553, 6561, 6563), pituitary tumours (ICD9 codes 1943, 2273, 2370), surgical history of terminal ileal resection (ICD9 code 4562), gastrectomy or small bowel bypass (ICD9 codes 430–4499), eating disorders (ICD9 codes 3071 and 30,750–30759), alcoholism (ICD9 codes 30,390–30393), regular use of gonadotropin-releasing hormone agonist, glucocorticoids, anticonvulsants, heparin, vitamin A and cytotoxic agents, were excluded from the study

From June 1, 2008, to May 31, 2018, 10,616 community-dwelling women were submitted to DXA and simultaneously completed the self-administered questionnaire regarding their smoking behaviours and their cohabiter’s (Fig. 1). Subjects underwent a DXA examination according to clinical criteria, as reported in Table 1. 3962 of them were classified as CS, and 6654 as non-smokers. Among the non-smokers, 873 were classified as PS and 5781 as NS, according to the smoking habit classification previously exposed (Fig. 1). Some clinical characteristics of CS, PS and NS are reported in Table 2. PS showed a lower mean age compared to NS and CS.

**Table 1:** Subjects included in the study classified according to the risk factor for osteoporosis for which dual-X-ray absorptiometry was prescribed according to Italian EAL

EAL criteria	Number of subjects
Major risk factor for men and women of any age	1241
Previous fragility fractures	556
Radiological finding of osteoporosis	912
Chronic therapies at risk of osteoporosis	0
Diseases at risk of osteoporosis	0
Major risk factors for postmenopausal women	3084
Family history of bone fracture at age lower than 75 years	3084
Body mass index < 19 kg/m^2^	0
Menopause at age lower than 45 years	0
Minor risk factors for postmenopausal women and men > 60 years	6291
Age > 65 years	5976
Family history of severe osteoporosis	432
Amenorrhoea > 6 months in premenopausal age	369
Inadequate calcium intake	4897
Vitamin D deficiency	5638
> 20 cigarettes/day	2627
Alcohol abuse (> 60 g/day)	868

*EAL* essential assistance levels

**Table 2:** Clinical characteristics of the study populations

	CS	PS	NS
Numbers	3962	873	5781
Mean age (years)	69.4 ± 10.4^a^	67.8 ± 11.6	69.4 ± 10.8^a^
BMI (kg/m^2^)*	27.1 ± 4.9	27.0 ± 5.0	27.0 ± 4.9
eGFR (ml/min/1.73m^2^)	77.6 ± 12.2	76.8 ± 11.9	77.3 ± 12.2
Op: controls	3309 (83.5): 653 (16.5)	727 (83.3): 146 (16.7)	4526 (78.3): 1225 (21.7)

Data are expressed as absolute number (percentage) or mean ± standard deviation for discrete and continuous variables, respectively. *CS* current smokers. *PS* passive smokers. *NS* never smokers. *BMI* body mass index. *eGFR* estimated glomerular filtration rate [17]. Op: subjects diagnosed with osteoporosis [22]. Controls: subjects with a lumbar, total hip or femoral T-score > − 2.5 and a personal history negative for clinical fragility fractures and/or anti-osteoporotic treatment. eGFR: estimated glomerular filtration rate

*After log-transformation

^a^Significantly different compared to PS (*p* < 0.01)

**Fig. 2:**
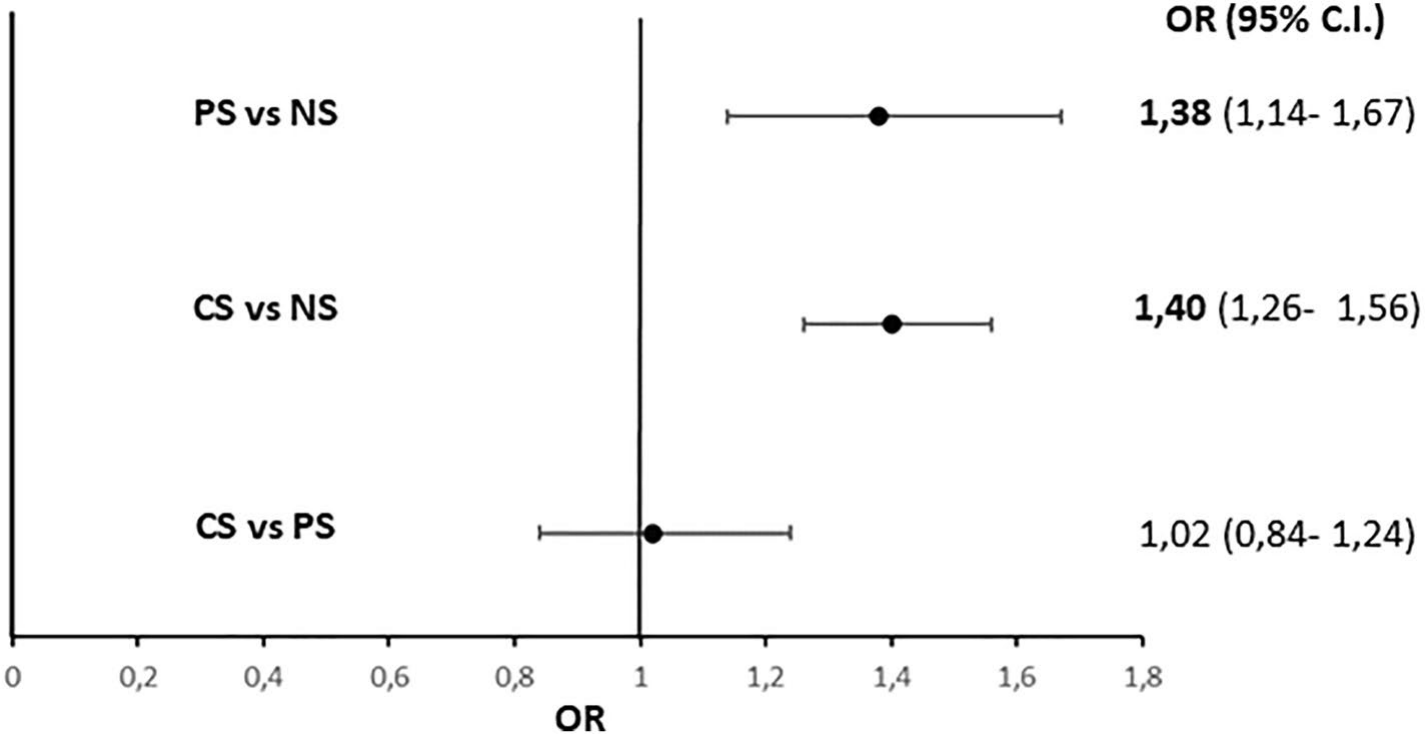
Risk of osteoporosis in the study population (current smoker and never smoker women). *CS* current smokers. *PS* passive smokers, *NS* never smokers, *OR* odds ratio; 95% C.I.: 95% confidence intervals. *analysis based on *n* = 10,206

In the entire study population, according to criteria previously reported, 8562 women (mean age 70.3 ± 10.2 yrs; BMI 27.0 ± 4.9 kg/m^2^; eGFR 77.7 ± 12.1 ml/min/1.73 m^2^) were diagnosed with Op. The remaining 2054 women who did not receive the Op diagnosis (mean age 64.9 ± 11.7 yrs; BMI 27.3 ± 5.1 kg/m^2^, eGFR 76.1 ± 12.3 ml/min/1.73 m^2^) were considered as controls. As depicted in Fig. 2, the Op prevalence in CS was significantly higher compared to NS [odd ratio (OR) 1.40 (95% CI: 1.26–1.56)]. Likewise, the Op prevalence in PS was significantly higher compared to NS [OR 1.38 (95% CI: 1.14–1.67)], but not different compared to CS [OR 1.02 (95% CI: 0.84–1.24)]. All these differences remained significant also after correction for age, BMI, eGFR, family history of bone fractures, inadequate calcium intake, vitamin D deficiency, and alcohol abuse as shown in Table 3.

**Table 3:** Odds ratio of osteoporosis according to smoke habit adjusted for major cofounding factors

Model	Beta	OR (95%CI)	*p*-value
Model 1			
Unadjusted			
PS vs*.* NS	0.32	1.38 (1.14–1.67)	0.001
Adjusted			
PS vs*.* NS	2.11	8.23 (5.40–12.57)	< 0.001
Age (for 1-year increase)	0.05	1.05 (1.04–1.06)	< 0.001
BMI (for 1 kg/m^2^ increase)	− 0.01	0.98 (0.97–0.99)	0.024
eGFR (for 1 ml/min/1.73 m^2^ increase)	− 0.01	1.00 (0.99–1.01)	0.35
Family history of bone fracture (yes vs*.* no)	0.07	1.07 (0.91–1.26)	0.41
Inadequate calcium intake (yes vs*.* no)	0.07	1.07 (0.92- 1.25)	0.36
Vitamin D deficiency (yes vs*.* no)	− 0.06	0.94 (0.82–1.07)	0.35
Alcohol abuse (yes vs*.* no)	− 0.12	0.88 (0.69–1.13)	0.33
Model 2			
Unadjusted			
CS *vs.* NS	0.34	1.40 (1.26–1.56)	< 0.001
Adjusted			
CS *vs.* NS	0.34	1.40 (1.26–1.57)	< 0.001
Age (1-year increase)	0.04	1.04 (1.04–1.05)	< 0.001
BMI (1 kg/m^2^ increase)	− 0.02	0.98 (0.97–0.99)	< 0.001
eGFR (for 1 ml/min/1.73 m^2^ increase)	− 0.01	0.99 (0.98–1.01)	0.45
Family history of bone fracture (yes vs*.* no)	0.12	1.01 (0.90–1.14)	0.85
Inadequate calcium intake (yes vs*.* no)	0.07	1.07 (0.96–1.19)	0.23
Vitamin D deficiency (yes vs. no)	− 0.07	0.93 (0.83–1.04)	0.21
Alcohol abuse (yes vs*.* no)	− 0.04	0.96 (0.78–1.17)	0.66
Model 3			
Unadjusted			
CS vs PS	0.02	1.02 (0.84–1.24)	0.86

*OR* odds ratio, *CI* confidence interval. *CS* current smoker, *PS* passive smoker, *NS* never smokers. Model 1: comparison between passive smokers and never smokers. Model 2: comparison between current smokers and never smokers. Model 3: comparison between current smokers and passive smokers. *BMI* body mass index. *eGFR* estimated glomerular filtration rate

Subjects undergoing DXA because of reported fractures were 250 (6.3%) among CS, 62 (7.1%) among PS and 244 (4.2%) among NS. CS presented a higher risk of fractures compared to NS [OR 1.58 (95% I.C. 1.27–1.83), *p* < 0.05], but not compared to PS [OR 0.88 (95% I.C. 0.66–1.17), *p* > 0.05]. In turn, PS presented a significant higher risk of fractured compared to NS [OR 1.68 (95% I.C. 1.28–2.20), *p* < 0.05]. No statistically significant difference was found in the prevalence of vitamin D deficiency among the three groups: subjects with vitamin D deficiency were 2127 (53.7%) in the CS group, 511 (58.5%) in PS and 3000 (51.9) in NS. Also, the prevalence of family history of bone fractures, inadequate calcium intake and alcohol abuse among the three study groups was not statistically significant.

## Discussion

The study results underline the presence of a significant association between passive smoking and Op in non-smoker community-dwelling women of European ancestry and suggest that CS and PS women have a similar increased risk of Op and fracture compared to NS.

Bone structure may be altered by exposure to smoke. In particular, increased levels of cross-links of the N-terminal collagen (NTx) were found in CS subjects [24], highlighting the changes in the composition of collagen fibres and in their cross-linking, which may affect the strength and resilience of the bone material. Therefore, any smoking-related increase in bone turnover can cause skeletal fragility [25].

ETS is generated by tobacco products’ combustion and it is a complex mixture of over 4000 compounds [2, 3]. These include more than 40 known or suspected human carcinogens, such as 4-aminobiphenyl,2-naphthylamine, benzene, nickel and various polycyclic aromatic hydrocarbons (PAHs) and N-nitrosamines. Furthermore, there are also several irritants in this mixture, such as ammonia, nitrous oxide, sulphur dioxide and aldehydes, and cardiovascular toxicants, such as carbon monoxide and nicotine [2, 3]. All these substances influence bone turnover through different pathways. In particular, experimental models showed that aldehydes can induce intense oxidative stress and inflammatory reactions. Excessive oxidative stress impairs osteoblast function by suppressing differentiation and inducing apoptosis. Additionally, oxidative stress also increased the responsiveness of osteoclast precursors to the receptor activator of nuclear factor-κB ligand (RANKL) signalling cascade and further stimulated the production of osteoclastogenic cytokines [26]. Meanwhile, PAHs suppress bone resorption by osteoclasts and bone synthesis by osteoblasts [27]. Another experimental model proved that nicotine administration can elevate RANKL and decrease osteoprotegerin levels in serum to stimulate osteoclast genesis. On the contrary, interrupting administration of nicotine to mice increased bone mass and decreased levels of the bone resorption markers [28].

The mechanisms underlying the association between passive smoke and Op are not well known, but its impact on bone mass may be even stronger in PS than CS, because of the mains and side stream smoke that composes the ETS mixture [29, 30]. Notably, side-stream smoke is formed from smouldering of cigarettes or other paraphernalia between puffs, while mainstream smoke is emitted at the mouthpiece during a puff, then exhaled by a smoker [6]. In addition, passive smoking accounts also for exposure to third-hand smoke. Third-hand smoke is defined as “residual tobacco smoke pollutants that remain on surfaces and in dust after tobacco has been smoked” [31]. In third-hand smoke, the exposure happens through dust ingestion, dermal absorption and inhalation due to residual tobacco smoke pollutants on surfaces such as floors, counters and walls. Moreover, residual tobacco smoke can persist even for months after smoking [31]. It is also clear that living in a geographical area with a high environmental air pollution can also affect the association between second- or third-hand smoke and BMD reduction [16].

Another element to consider is represented by the half-time of cotinine, the predominant catabolite of nicotine usually used as biomarker for exposure to tobacco smoke that has been found to be significantly higher in serum, urine and saliva of PS compared to CS [31]. The serum cotinine has also been demonstrated to negatively associate with the vitamin D concentration in community-dwelling population [32, 33], partially clarifying the reasoning behind our result. In this regard, it must be noted that our population is not representative of a community-dwelling one, since it has been selected according to EAL criteria to be subjected to DXA, accounting for a high prevalence of vitamin D deficiency among the three groups.

Consistent with our results, the association between passive smoking and low BMD was observed in different ages and ethnic groups worldwide [31, 32, 34]. In two retrospective studies, passive smoking has been inversely associated with phalangeal BMD in adults [35] and with total hip and femoral neck BMD in 154 premenopausal women [36]. In a prospective study, the exposure to passive smoking in childhood was an important determinant of reduced BMD, density and strength indices measured 28 years later in adulthood [37].

One further last observation is represented by the tangled interaction between ETS, BMI and Op. The latter are notoriously intertwined with one another: BMI is indeed inversely associated with Op risk [38], whereas smoke presents with a direct association [39]. The joint effect of ETS and BMI is a well-known pattern for the occurrence and the worsening of other conditions, such as asthma [40, 41], tumour and cancer-related death [42], hypertension [43], gout [44] and abnormalities in glucose metabolism [45]. Our results confirm that Op risk related to ETS dramatically increases when BMI is included in the multivariate model in PS, but not to the same extent in CS. The low dose and nonmonotonicity of some chemical compounds from side smoke in PS can be one reason for the different result observed in PS and CS [46]. It must also be considered that side stream smoke from passive smoking is a low-temperature oxygen-poor environment that contains higher concentrations of ammonia and nitric oxides, which are smaller than the ones produced during the act of smoking [47]. Further studies will be needed to better clarify these observations.

Some strengths and limits of this study are strictly related to the use of administrative databases [34]. Their use warrants a high sample size with reasonable costs, and, in effect, we enrolled a large and very homogeneous population. In addition, the sensitivity and specificity of Op diagnosis based on administrative health database are very affordable, especially when the prescription of anti-osteoporotic drugs is used as diagnostic criteria. Finally, the study protocol excludes the more prevalent causes of secondary Op. All data collected for this study are integral part of the usual clinical management of the adult subjects registered in the Italian National Health System. On the other hand, the use of administrative data does not allow evaluating if the anatomical site of DXA examination showing pathological BMD finding (i.e. lumbar, femoral or ulnar site) influences the study results, or the number of cigarette smoked per day. We are also not able to obtain data regarding some factors that can influence the BMD (such as vitamin D status, physical activity, dietary habits and socio-economics factors) of enrolled subjects, or the duration of the exposure to second- or third-hand smoke. Moreover, we used data obtained with different types of DXA machines, albeit all measurements were carried out in clinical centres affiliated with the Italian Health National System (in Italian *Sistema Sanitario Nazionale*) and therefore each clinical centre is subjected to unique quality controls (International Organization for Standardization—ISO 9001). Another limit of the study is its cross-sectional and retrospective nature, which impairs establish cause–effect relationships between passive smoking and Op. Actually, to our knowledge, this is the first study based on women population of European ancestry.

A double-edged sword may be represented by the generalizability of the study results. Indeed, our study population is not representative of all community-dwelling women, but only of those undergoing DXA evaluation of BMD. The examined women are just representative for those that already present a higher risk of Op and fragility fractures, being selected according to the Italian Ministry of Health prevention plan, based in turn on the WHO criteria. On the other hand, our selection criteria provided a population that presented already with a higher risk of Op than the general public and it miss to recognize other significant differences among the three groups, such as BMI and vitamin D deficiency. Nevertheless, the high prevalence of Op provides also with the opportunity of studying specific risk factors, such as passive smoke. The selection criteria and the high environmental air pollution are responsible for the high prevalence of Op observed in the entire study population (more than 80%).

In conclusion, despite these limits, our study results suggest, that PS showed an Op risk similar to the one observed in CS and significantly higher to the risk observed in NS. Finally, active and passive smoking could be two equivalent Op risk factors, but further prospective studies, using different study methodologies, are desirable to expand our knowledge in this intriguing and epidemiological very important research setting. This study sends the provocative message that the inclusion of exposure to ETS as an Op risk factor is needed and that it may affect the screening programs.

## Declarations

### Research involving human and animal participants

All procedures performed in this study, which involves human participants, were in accordance with the ethical standards of the institutional and/or national research committee and with the 1964 Helsinki Declaration and its later amendments or comparable ethical standards.
